# Facebook Recruitment of Vaccine-Hesitant Canadian Parents: Cross-Sectional Study

**DOI:** 10.2196/publichealth.6870

**Published:** 2017-07-24

**Authors:** Jordan Lee Tustin, Natasha Sarah Crowcroft, Dionne Gesink, Ian Johnson, Jennifer Keelan, Barbara Lachapelle

**Affiliations:** ^1^ School of Occuaptional and Public Health Ryerson University Toronto, ON Canada; ^2^ Dalla Lana School of Public Health University of Toronto Toronto, ON Canada; ^3^ Public Health Ontario Toronto, ON Canada; ^4^ Laboratory Medicine and Pathobiology University of Toronto Toronto, ON Canada; ^5^ Concordia University of Edmonton Edmonton, AB Canada; ^6^ Toronto Public Health Toronto, ON Canada

**Keywords:** immunization, vaccination, social media, Canada, parents

## Abstract

**Background:**

There is concern over the increase in the number of “vaccine-hesitant” parents, which contributes to under-vaccinated populations and reduced herd immunity. Traditional studies investigating parental immunization beliefs and practices have relied on random digit dialing (RDD); however, this method presents increasing limitations. Facebook is the most used social media platform in Canada and presents an opportunity to recruit vaccine-hesitant parents in a novel manner.

**Objective:**

The study aimed to explore the use of Facebook as a tool to reach vaccine-hesitant parents, as compared with RDD methods.

**Methods:**

We recruited Canadian parents over 4 weeks in 2013-14 via targeted Facebook advertisements linked to a Web-based survey. We compared methodological parameters, key parental demographics, and three vaccine hesitancy indicators to an RDD sample of Canadian parents. Two raters categorized respondent reasons for difficulties in deciding to vaccinate, according to the model of determinants of vaccine hesitancy developed by the World Health Organization’s Strategic Advisory Group of Experts on Immunization.

**Results:**

The Facebook campaign received a total of 4792 clicks from unique users, of whom 1696 started the Web-based survey. The total response rate of fully completed unique Web-based surveys was 22.89% (1097/4792) and the survey completion rate was 64.68% (1097/1696). The total cost including incentives was reasonable (Can $4861.19). The Web-based sample yielded younger parents, with 85.69% (940/1097) under the age of 40 years as compared with 23.38% (408/1745) in the RDD sample; 91.43% (1003/1097) of the Facebook respondents were female as compared with 59.26% (1034/1745) in the RDD sample. Facebook respondents had a lower median age of their youngest child (1 year vs 8 years for RDD). When compared with the RDD sample, the Web-based sample yielded a significantly higher proportion of respondents reporting vaccines as moderately safe to not safe (26.62% [292/1097] vs 18.57% [324/1745]), partially or not at all up-to-date vaccination status of youngest child (22.06% [242/1097] vs 9.57% [167/1745]), and difficulty in making the decision to vaccinate their youngest child (21.06% [231/1097] vs 10.09% [176/1745]). Out of the Web-based respondents who reported reasons for the difficulties in deciding to vaccinate, 37.2% (83/223) reported lack of knowledge or trust due to conflicting information and 23.8% (53/223) reported the perception of the risk of the adverse effects of vaccines being higher than the risk of disease acquisition.

**Conclusions:**

We successfully recruited a large sample of our target population at low cost and achieved a high survey completion rate using Facebook. When compared with the RDD sampling strategy, we reached more vaccine-hesitant parents and younger parents with younger children—a population more likely to be making decisions on childhood immunizations. Facebook is a promising economical modality for reaching vaccine-hesitant parents for studies on the determinants of vaccine uptake.

## Introduction

### Background

Immunization is one of the most important accomplishments in the global fight against infectious diseases. In Canada, vaccines have saved more lives than any other public health intervention [1]. Despite this success, a 2011 Canadian national survey on immunization coverage reported sub-optimal coverage rates for recommended childhood vaccinations [2]. This low coverage among Canadian children is of concern as vaccine-preventable diseases (VPDs) endemic in other parts of the world could be imported into Canada and lead to outbreaks due to transmission among unvaccinated or under-vaccinated individuals in low coverage areas [3]. Measles is still common in developing countries and remains one of the leading causes of death in young children [4]. The import of measles into Canada was made evident with several recent outbreaks [3]. For example, in 2011, the province of Québec reported the largest North American outbreak of measles since 2002, with 776 cases as compared with the usual annual average of 0 to 2 cases [5,6]. In 2013, there were nine measles outbreaks in Canada with more than half of the cases (42/71) linked to one outbreak in a non-immunizing community in Alberta [7]. In March 2014, the Public Health Agency of Canada (PHAC) released a public health notice, warning Canadians of unusually high numbers of measles cases in five Canadian provinces [8,9]. In 2015, another notice was released because of outbreaks in Ontario and Quebec and the multi-state measles outbreak in the United States [10]. Outbreaks of VPDs such as measles are an imminent threat to Canadians, and experts have suggested that lower vaccine coverage rates are an “impending crisis” [11].

Vaccine hesitant individuals are a “heterogeneous group in the middle of a continuum ranging from total acceptors to complete refusers” [12]. These individuals are of interest as they are undecided about vaccination and may decide to accept, refuse, or delay all or some vaccines for themselves or their children [12]. A recent systematic review by Larson et al (2014) on vaccine hesitancy found that factors affecting vaccine hesitancy include confidence in the vaccine or the provider, complacency regarding the need for or effectiveness of the vaccine, and convenience in terms of access to health care or vaccines [12]. The Strategic Advisory Group of Experts Working Group (SAGE WG) on Immunization recently built on this definition by organizing vaccine hesitancy around three domains: contextual influences such as socio-economic barriers or communications via media/social media, individual/social group influences such as personal knowledge or perceptions of risk, and vaccinations and vaccination-specific issues such as vaccination schedules or characteristics of the vaccines [12-14].

There is a critical need to better understand the factors underlying vaccine hesitancy in Canada in order to implement interventions to help parents in their decision to vaccinate and increase vaccine coverage. Random digit dialing (RDD) surveys have historically been the “Gold Standard” in the collection of Canadian immunization study data. However, Statistics Canada reports that more Canadian households are abandoning their traditional landline telephones; the number of households with landlines has fallen from 66% of households in 2010 to 56% in 2013 [15]. In the province of Quebec, only 43% of households reported having a landline [15]. In contrast, Internet use has been steadily increasing over the years and as of 2010, 80% of Canadians 16 years of age and older use the Internet at home at least once per day [16]. In 2012, this increased to 83% [17]. In addition, the majority (58%) of Internet users are using social media, including over 86% of those under the age of 35 [16], that is, those in their peak reproductive, childbearing, and small-child-rearing years. Concerns have been emerging in the public health community that parental fears regarding childhood vaccines are growing, largely due to rapid sharing of misinformation and the increasing expression and empowerment of anti-vaccine communities and activists on social media [11,18]. Therefore, recruiting via social media platforms for Web-based surveys should be investigated as a viable alternative or complement to RDD to reach self-selecting higher risk populations, such as vaccine-hesitant parents. Alshaikh et al (2013) conducted a systematic review of articles using social media for health research and reported that despite the risk of sampling bias, social media platforms are a useful tool in health research [19]. Furthermore, several recent studies have reported the success of Facebook as a viable, rapid, and cost-effective platform for targeted recruitment of specific populations such as pregnant women, unvaccinated women, parents, young adults and smokers [20-24].

### Objective

This study aims to explore the effectiveness of Canada’s most popular social media platform, Facebook, as a tool to reach vaccine-hesitant parents, and it will explore the differences in key parental demographics and vaccine hesitancy indicators between a Facebook survey of recruits and the most recent RDD survey of the Canadian population [25]. To date no study has investigated the value of social media recruitment versus traditional RDD household recruitment in the study of parental immunization practices and beliefs.

## Methods

### Study Design

In this observational study, we used two datasets that included data on parental immunization beliefs and practices collected from Canadian parents via two different cross-sectional methods. The inclusion criteria for both populations were as follows: (1) over 18 years of age, (2) having at least one child under 18 years, (3) living in Canada, and (4) able to respond to questions in English or French.

Population-based data were de-identified and extracted from a survey collected by a research company contracted by PHAC. During a period of three weeks in March 2011, the researchers randomly selected a sample of Canadian households with a landline via RDD and administered a telephone survey in French or English. The telephone survey consisted of questions on demographics and Canadian parents’ knowledge, awareness, attitudes, and behaviors related to immunization [25]. The researchers attempted contact with each household in the sample 8 times prior to retiring the phone number [25]. The average time to complete the survey was 18 minutes and 30 seconds [25]. Researchers of the RDD sample reported a participation rate of 23.43% (7898/33,698) and a total cost of Can $163,398. The average cost per completed survey was Can $93.64.

The Web-based survey comprised primary data collected from self-selected respondents recruited via the social media platform, Facebook. Facebook has been reported as the most popular social media platform in Canada, with more than half of the population logging into Facebook at least once per month, and daily Facebook usage reported as higher than global and US averages [26,27]. The Web-based semistructured survey was available in French and English and contained questions similar to the RDD survey on demographics, parents’ knowledge, awareness, attitudes, and behaviors related to immunization. Trusted website links with reliable information on childhood immunizations appeared immediately after terminating or completing the survey to ensure there was no prior influence on the respondents. We piloted the survey with a convenience sample of the primary researcher’s “Facebook friends” and a snowball sample of the friends’ “Facebook friends” who met the inclusion criteria.

The Web-based survey was set to automatically terminate if the respondents did not provide informed consent or did not meet eligibility criteria. We used a Canadian Web-based survey company (now owned by an American company), Fluid Surveys, to capture the survey data. Fluid Surveys stored all of its data in Canada and used the latest in firewall and encryption technology to protect private information. We exported, encrypted, and password protected the survey responses and did not collect any identifying information on respondents. Upon completion of the survey, respondents were eligible to participate in a draw with an estimated 1 in 90 chance (based on an estimated sample size of 800 respondents participating in the draw) to win an iPad mini (value of Can $375). We kept all email addresses of participating respondents confidential and destroyed them at the end of the draw. We obtained ethical approval from University of Toronto’s Office of Research Ethics (REF# 29309).

### Recruitment

We displayed Facebook advertisements on the News Feeds of Facebook users whose profiles matched the following inclusion criteria: (1) located in Canada, (2) 18 years or older, (3) parent of a child aged 0 to 15 years, and (4) displaying a profile in English or French. Our advertisements did not target parents with children aged 16-19 years as they are self-consenting to immunization and their inclusion would significantly increase the target audience and dilute our advertisements; however, they would be included if they had younger children. Facebook determines users’ location based on information in their timeline, verified by their Internet Protocol (IP) address and by examination of the user’s friends’ locations [28]. A user’s age was determined by their year of birth, required by Facebook for all personal accounts [28]. Parents were identified based on activity or information on their timelines and language was determined from the language used in their profiles [28].

**Figure 1:**
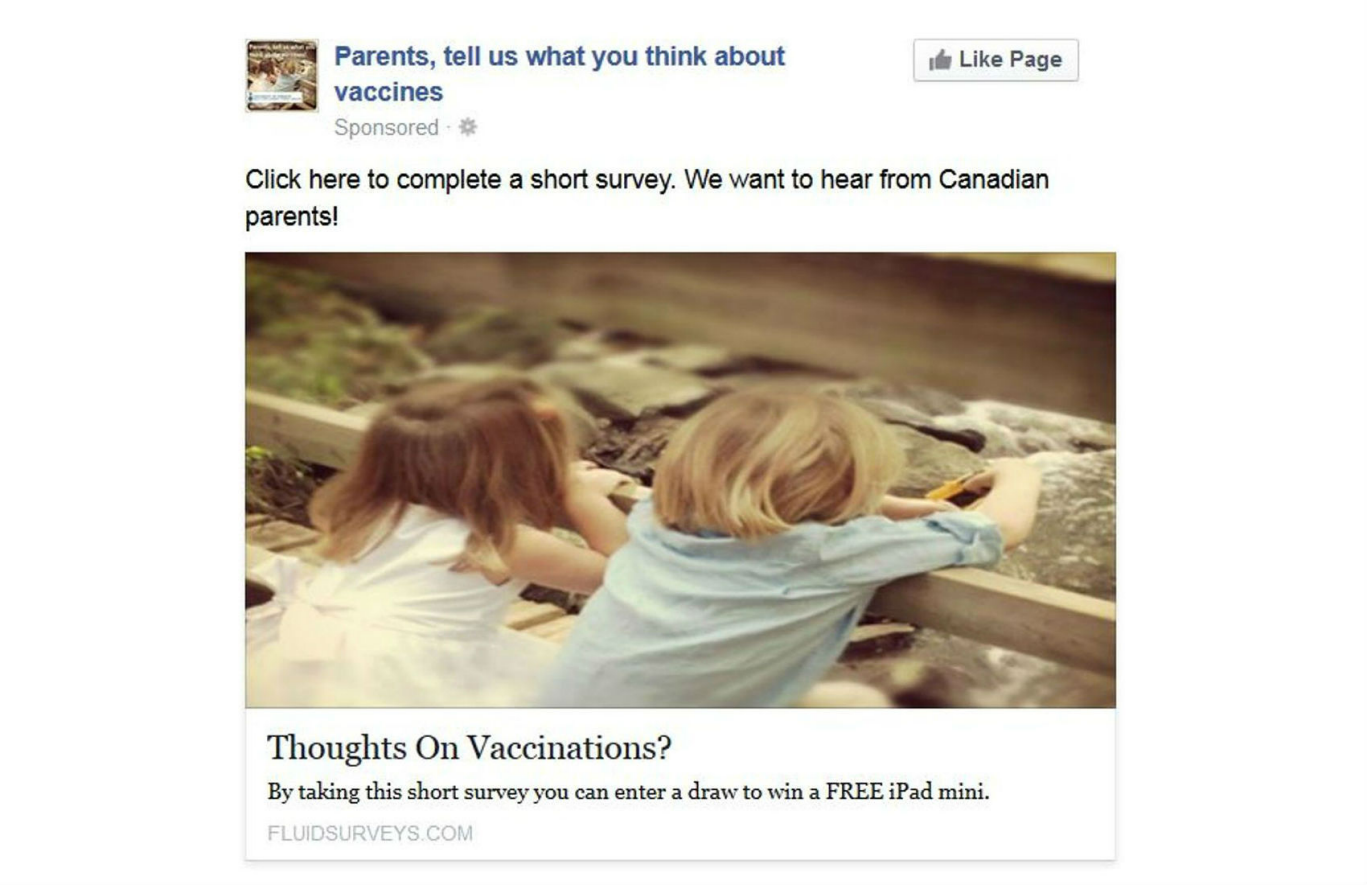
Facebook advertisement A in the English campaign.

**Figure 2:**
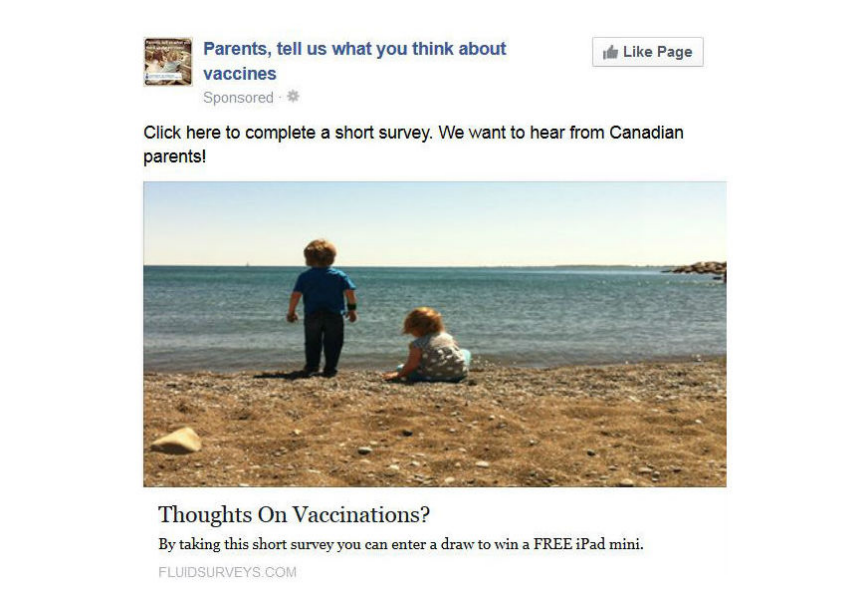
Facebook advertisement B in the English campaign.

**Figure 3:**
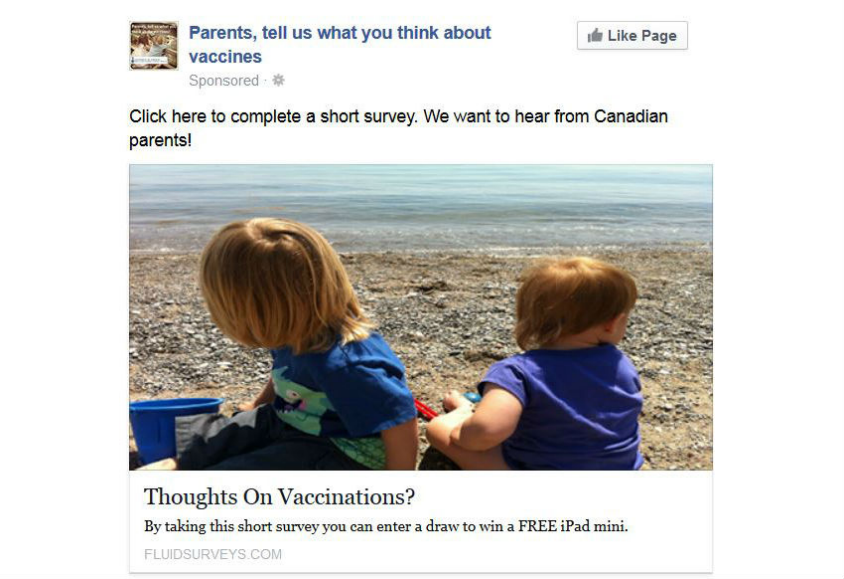
Facebook advertisement C in the English campaign.

The optimal delivery mechanism of advertisements on Facebook is determined by many factors such as the target audience, the marketplace competition, the bid, and the advertisement’s performance history [29]. Facebook provides the option of being charged each time the advertisement is displayed, that is, cost per thousand impressions (CPM) or each time the advertisement is clicked (CPC) [29]. We chose to pay based on CPM as Facebook ensures the advertisement will be optimized to the people most likely to click on your advertisement (eg, most active and engaging users) and remains in the optimal bid range. In addition, Facebook paces the rate at which the advertisement is displayed based on the budget, goal, and period of time specified [29]. We set a goal to reach a minimum of 800 participants based on power calculations and our budget for survey incentives. We began with a lifetime budget of Can $1500 over a period of one month, as this would grant us access to a Facebook consultant. At the time of our advertisement launch, there was a potential to reach 300,000 Canadian parents on Facebook (260,000 English users and 40,000 French users). Therefore the money was allocated based on this distribution with approximately 85% of the budget allocated to the English campaign. Fifty dollars (Can $) gifted by Facebook was later added to the French campaign budget. The Facebook advertisement campaign was launched on December 12, 2013, at 14:00 and ended on January 11, 2014, at 14:00. Three different images were used in our advertisement (Figures 1-3).

Facebook provided several advertisement statistics such as the number of clicks (eg, likes, comments, click for our Facebook page, and click for our Web-based survey), the number of impressions (placements on users’ News Feeds), the CPM, and the CPC. Based on these statistics, Facebook optimized the advertisements with the highest click-through rate (CTR) (the number of clicks received/number of impressions) to serve the most users. We removed advertisements that fell below the Facebook average CTR of 1-1.5 % from the campaign.

The objective of the campaign was for targeted Facebook users to click on the advertisement linked to our secure Web-based survey. Users could also be directed to our Facebook page titled “Parents, tell us what you think about vaccines” by clicking on the advertisement’s profile user as opposed to the link. We provided further information on the study and links to the survey on our official Facebook Page.

### Statistical Analysis

#### Campaign and Recruitment

We investigated methodological parameters on the number of impressions, the number of clicks, demographics of users who clicked the advertisement, timelines of data collection, and the costs for both the English and French campaigns. The response rate calculation for the Web-based sample is a derivation from definitions provided by the American Association for Public Opinion Research [30] and is the number of completed surveys divided by the total number of surveys (completed, partially completed, and terminated), plus the remaining unique clicks of unknown eligibility.

#### Respondent Characteristics and Vaccine Hesitancy

We validated Web-based sample data for single questionnaire response and accuracy of eligibility criteria by verifying IP addresses and demographic information. We conducted univariate analyses for the Web-based and the RDD samples on individual level variables for respondent characteristics and vaccine hesitancy indicators. Respondent characteristics included age group, sex, income and education level, median age of youngest child, birthplace, and place of residence. We investigated vaccine hesitancy using three indicators: perception of safety of childhood vaccinations, measured on a 7-point scale from “Not safe” (1) to “Moderately Safe” (4) to “Extremely Safe” (7); vaccination status of youngest child was classified as “Completely up-to-date” or “Partially or Not at all up-to-date;” and difficulty in making the decision to vaccinate (or not vaccinate) their youngest child, measured as “Very Easy,” “Easy,” “Difficult,” or “Very Difficult.” We conducted all descriptive analyses using Microsoft Office Excel 2007 and SAS Version 9.3 (SAS Institute Inc.).

A primary and secondary rater independently coded qualitative data from the Web-based survey on the difficulties in deciding to vaccinate youngest child according to the SAGE model of determinants of vaccine hesitancy [13]. Two raters independently coded all responses with a high level of agreement (percent agreement>90%). Discrepancies were resolved via consensus to reach 100% agreement. The raters could not code the pre-categorized open-ended responses from the RDD data. However, the raters classified the pre-coded categories according to best fit in the SAGE model. The raters conducted all qualitative analyses with NVivo 10 software (QSR International).

## Results

### Campaign and Recruitment

**Figure 4:**
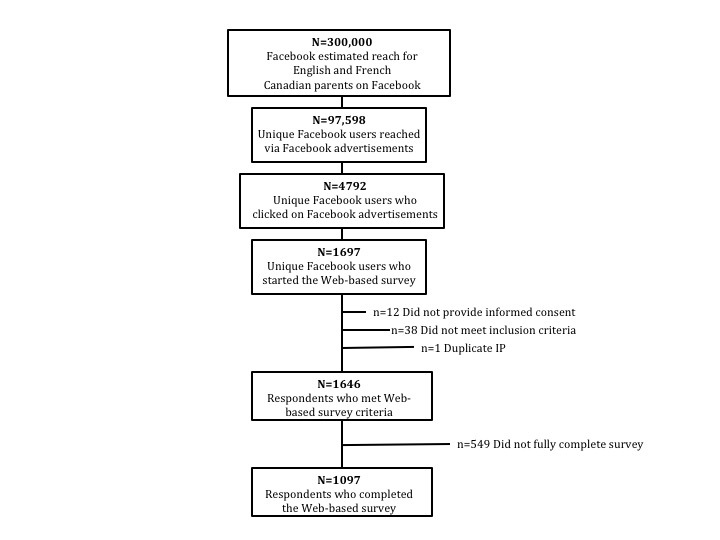
Facebook advertisement recruitment.

During the one-month campaign, our advertisements made 280,485 impressions yielding 8557 total clicks on our advertisements. The overall campaign CTR was 3.05% (8557/280,485), with the English campaign yielding a higher click rate of 3.57% (7981/223,637) as compared with 1.01% (576/56,848) for the French campaign. Over 75% (215,770/280,485) of the impressions were among women. Women aged 25-34 years were reached the most, with 39.15% (109,808/280,485) of the overall impressions. Thus, the majority (87.05% [7449/8557]) of the clicks on the advertisements were also women, with the highest average CTR of 2.82% (159/12,410 in the French campaign and 1818/41,804 in the English campaign) among women aged 35-44 years, followed closely by an average CTR of 2.59% among women aged 45-54 years (45/3008 in the French campaign and 261/7080 in the English campaign) and 2.53% among women aged 25-34 years (107/13,212 in the French campaign and 4111/96,596 in the English campaign). In terms of unique Facebook users, our campaign reached 32.53% (97,598/300,000) of our target population on Facebook, with 4.91% (4792/97,598) clicking on the advertisement. Out of the 4792 unique clicks on our advertisements, 35.41% (1697/4792) started the survey. Only fully completed surveys were counted as part of our sample, resulting in 1097 unique respondents. Thus, the response rate was 22.89% (1097/ 4792) and the survey completion rate was 64.68% (1097/1696), with very little missing data (Figure 4). The average time taken to complete the survey was 17 minutes.

**Figure 5:**
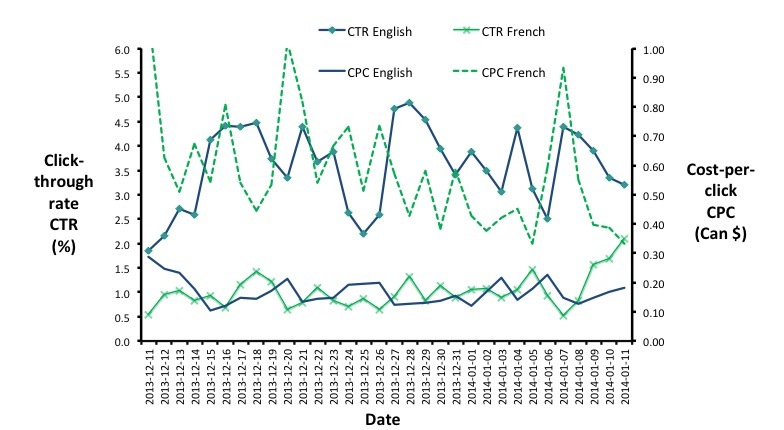
Daily click rates as compared with the cost per click for all campaigns from December 11, 2013, to January 11, 2014.

Advertisement success varied by language and image displayed. All advertisements produced clicks; however, advertisement A (Figure 1) produced the highest reach and click-through rate and had the lowest cost (Table 1). The CTR was consistently higher over time and the CPC consistently lower for the English campaign as compared with the French campaign. CTRs and CPCs were variable over time for both campaigns; however, the English campaign experienced a substantial drop in the CTR during the holidays from December 23 to 25, 2013. In periods of CTR decrease, there was a corresponding increase in CPC (Figure 5). For the English campaign, the average cost per 1000 impressions (CPM) was Can $5.59 and Can $5.28 for the French campaign. Translated into CPC, the English campaign cost an average of Can $0.16 and the average for the French campaign was Can $0.52. The total research cost was Can $4,861.19 (Can $1500 campaign cost – Can $50 Facebook credit + Can $3361.19 incentives cost).

**Table 1:** Facebook advertisement statistics.

Campaign		Reach (No. of unique Facebook users)	No. of impressions	No. of clicks	CTR^b^ (%)	No. of unique clicks	Unique CTR (%)	Average cost per CPM^c^ (Can $)	Average CPC^d^ (Can $)	Average cost per unique click (Can $)
**English** **advertisements**										
	A	74,572	153,217	5767	3.76	3346	4.49	5.39	0.14	0.25
	B	38,643	51,647	1778	3.44	1189	3.10	6.05	0.18	0.26
	C	16,919	18,773	436	2.32	368	2.18	5.98	0.26	0.30
										
**French** **advertisements^a^**										
	A	15,767	36,327	393	1.08	338	2.14	5.18	0.48	0.56
	B	9178	15,891	150	0.94	128	1.40	5.39	0.57	0.67
	C	3811	4630	33	0.71	33	0.87	5.63	0.79	0.79

^a^French advertisements B and C were removed from the campaign on January 3, 2014.

^b^CTR: click-through rate.

^c^CPM: cost per impression.

^d^CPC: cost per click.

### Respondent Characteristics

Of the respondents in the Web-based survey and the population-based RDD survey, 91.89% (1008/1097) and 83.61% (1459/1745), respectively were born in Canada. The distribution across place of residence was similar, except that the Web-based sample had a lower proportion of respondents from Québec as compared with the RDD sample (10.94% [120/1097] vs 24.47% [427/1745) and a higher proportion of respondents from Alberta (23.61% [259/1097] vs 11.46% [200/1745]). The age distribution differed, with 85.69% (940/1097) of the Web-based survey respondents under the age of 40 years as compared with 23.38% (408/1745) in the RDD sample; however, the age for 37.99% (663/1745) of the RDD respondents is not known. For both samples, the median number of children was two (IQR 1.0) with the median age of the youngest child at 1 year (IQR 1.50) in the Web-based sample and 8 years (IQR 10.0) in the RDD sample. The Web-based sample had 91.43% (1003/1097) female respondents as compared with 59.26% (1034/1745) in the RDD sample. Both samples had similar distribution of education and income level, with almost half of the respondents completing some level of higher education, following the education distribution of Canadian adults [31], and the majority lying close to or above the 2012 median total household income for Canadian families of $74,540 [32] (Table 2).

### Vaccine Hesitancy

Of the respondents from the Web-based survey, 26.62% (292/1097) perceived childhood immunizations to be not safe to moderately safe as compared with 18.57% of the RDD sample (324/1745), 22.06% (242/1097) reported the vaccination status of their youngest child to be not up-to date compared to 9.57% (167/1745) in the RDD sample, and 21.1% (231/1097) of the Web-based sample reported the decision to vaccinate their youngest child to be difficult or very difficult as compared with 10.09% (176/1745) in the RDD sample. In the Web-based sample, more than half of those not up-to-date reported that their youngest child had not received any vaccinations (126/242), with 6.3% (n=8/126) reporting the child was too young for vaccinations (Table 3). In total, 20.2% (49/242) of the respondents with their youngest child not up-to-date in terms of vaccinations reported concerns over autism or sudden infant death syndrome as important reasons for deciding not to vaccinate their youngest child.

Of those who found the decision difficult or very difficult, 54.8% (125/228) of the Web-based sample and 36.4% (64/176) of the RDD sample reported their youngest child to be not-up-to date. No significant trends were found when stratifying by parental age, parity, and sex.

In the Web-based sample, 79.8% (178/223) of the reasons for difficulty in their decision making were reported as individual and group influences with knowledge/awareness of vaccination information reported as the most important determinant for 35.4% (40/113) of those with their youngest child up-to-date and 39.1% (43/110) of those who reported their child as not-up-to-date (Table 4). In terms of knowledge, the majority reported difficulties with too much controversial or contradicting information and not enough unbiased or trustworthy information. The second highest determinant reported was the perception of the risks/benefits of vaccination, reported by 23.9% (27/113) of parents with an up-to-date child and 23.6% (26/110) of those whose children were not up-to-date. Most struggled with the risk of adverse effects or side effects versus the risk of acquiring the disease, where 23% (12/53) specifically expressed concern over the risk of autism. Approximately 6.0% (7/113) in the up-to-date and 9.1% (10/110) in the not-up-to-date group reported pressure from society, family/friends, or physicians to vaccinate or not. To a lesser extent, other individual or group influences included personal experience or knowledge of someone who subsequently experienced side effects or developed autism after vaccination, distrust of the government, and belief that vaccines are not necessary for health. Vaccine or vaccination specific issues were reported as reasons in 12.1% (27/223) of the sample. The majority in both groups reported issues with the vaccination schedules in terms of multiple vaccines or age of vaccination, followed by issues with lack of research or testing of new vaccines. Approximately 8% (18/223) of the reasons were reported as contextual, with respondents reporting distrust of the pharmaceutical industry, controversial coverage or fear mongering by the media, and forced vaccination as a result of mandatory vaccination policies in schools. Based on the pre-coded categories in the RDD sample, the majority of both up-to-date and not up-to-date parents also reported perception of risks/benefits and knowledge/awareness as the most important reasons why their decision to vaccinate was difficult or very difficult.

**Table 2:** Respondent demographic characteristics.

Characteristic		Web-based survey (N=1097)			Population-based RDD survey, un-weighted (N=1745)		
		n	%	95% CI	n	%	95% CI
**Age group (years)**								
	Under 30	395	36.01	33.20-38.88	57	3.27	2.51-4.18
	30-34	356	32.45	29.73-35.27	129	7.39	6.23-8.69
	35-39	189	17.23	15.08-19.55	222	12.72	11.22-14.35
	40-44	96	8.75	7.19-10.53	244	13.98	12.41-15.67
	45 and over	56	5.10	3.92-6.53	430	24.64	22.66-26.71
	Unknown	5	0.46	0.17-1.01	663	37.99	35.74-40.29
**Sex**								
	Male	80	7.29	5.86-8.95	711	40.74	38.46-43.06
	Female	1003	91.43	89.66-92.98	1034	59.26	56.94-61.54
	Unknown	14	1.28	0.73-2.08	-	-	
**Education level**								
	Did not graduate high school	25	2.28	1.51-3.30	83	4.76	3.83-5.83
	High school diploma	147	13.40	11.48-15.51	275	15.76	14.11-17.53
	Trade or vocational school	286	26.07	23.54-28.73	514	29.46	27.35-31.63
	Some university	110	10.03	8.35-11.91	144	8.25	7.02-9.61
	Bachelor’s degree	277	25.25	22.75-27.89	404	23.15	21.22-25.17
	Professional certification	123	11.21	9.45-13.18	97	5.56	4.56-6.71
	Graduate degree	101	9.21	7.60-11.03	221	12.66	11.17-14.29
	Unknown	28	2.55	1.74-3.62	7	0.40	0.18-0.86
**Income level (Can $)**								
	Under $30,000	85	7.75	6.27-9.44	157	9.00	7.22-10.41
	$30,000-$70,000	236	21.51	19.16-24.02	522	29.91	27.80-32.09
	$70,000-$79,999	92	8.39	6.85-10.14	125	7.16	6.02-8.45
	$80,000-$119,999	316	28.80	26.18-31.54	381	21.83	19.94-23.82
	Over $120,000	256	23.34	20.90-25.91	374	21.43	19.56-23.41
	Unknown	112	10.21	8.52-12.11	186	10.66	9.28-12.17
**Province or Territory of residence**								
	British Columbia	160	14.59	12.59-16.77	175	10.03	8.68-11.51
	Alberta	259	23.61	21.17-26.19	200	11.46	10.03-13.02
	Saskatchewan	95	8.66	7.10-10.44	101	5.79	4.76-6.96
	Manitoba	42	3.83	2.80-5.09	96	5.50	4.50-6.65
	Ontario	336	30.63	27.95-33.41	486	27.85	25.79-29.99
	Québec	120	10.94	9.19-12.89	427	24.47	22.50-26.53
	New Brunswick	26	2.37	1.59-3.40	62	3.55	2.76-4.50
	Nova Scotia	31	2.83	1.96-3.94	70	4.01	3.16-5.01
	Prince Edward Island	5	0.46	0.17-1.01	30	1.72	1.18-2.42
	Newfoundland	16	1.46	0.87-2.31	46	2.64	1.96-3.47
	Yukon	3	0.27	0.07-0.74	15	0.86	0.50-1.38
	Northwest Territories	2	0.18	0.03-0.60	23	1.32	0.86-1.94
	Nunavut	-	-	-	14	0.80	0.46-1.31
	Unknown	2	0.18	0.03-0.60	-	-	-
**Birthplace**								
	Canada	1008	91.89	90.16-93.39	1459	83.61	81.82-85.29
	Outside of Canada	61	5.56	4.32-7.04	286	16.39	14.71-18.18
	Unknown	28	2.55	1.74-3.62	-	-	-

**Table 3:** Respondent perception of safety of childhood vaccination, vaccination status of youngest child, and difficulty in making the decision to vaccinate youngest child.

Characteristic		Web-based survey (N=1097)			Population-based RDD survey, Un-weighted (N=1745)		
		n	%	95% CI	n	%	95% CI
**Perception on safety of childhood immunizations**								
	1-Not at all safe	49	4.47	3.36-5.82	43	2.46	1.81-3.28
	2	48	4.38	3.28-5.71	24	1.38	0.90-2.01
	3	64	5.83	4.56-7.34	50	2.87	2.16-3.73
	4-Moderately safe	131	11.94	10.12-13.96	207	11.86	10.41-13.44
	5	134	12.22	10.38-14.25	275	15.76	14.11-17.53
	6	338	30.81	28.13-53.59	500	28.65	26.57-30.81
	7-Extremely safe	327	29.81	27.16-32.57	630	36.10	33.87-38.38
	Unknown	6	0.55	0.22-1.13	16	0.92	0.54-1.45
**Vaccination status of youngest child**								
	Completely up-to-date	851	77.58	75.03-79.97	1552	88.94	87.40-90.35
	Somewhat-up-to-date or not at all up-to-date	242	22.06	19.68-24.59	167	9.57	8.26-11.02
	Unknown	4	0.36	0.12-0.88	26	1.49	0.99-2.15
**Difficulty in making the decision to vaccinate youngest child**								
	Very easy	624	56.88	53.94-59.79	642	36.79	34.55-39.07
	Easy	234	21.33	18.98-23.83	914	52.38	50.03-54.72
	Difficult	152	13.86	11.91-16.00	131	7.51	6.34-8.82
	Very difficult	79	7.20	5.78-8.85	45	2.58	1.91-3.41
	Unknown	8	0.73	0.34-1.38	13	0.74	0.42-1.24

**Table 4:** Web-based survey respondent reasons for difficulty in deciding to vaccinate youngest child by youngest child vaccination status.

SAGE Model determinant of vaccine hesitancy		Vaccination status of youngest child					
		Up-to-date		Not up-to-date			
		n	%	n	%	Total	Total %
**Contextual influences**								
	Communication and media environment	3	2.7	1	0.9	4	1.8
	Influential leaders, gatekeepers, and anti- or pro-vaccination lobbies	1	0.9	-	-	1	0.5
	Pharmaceutical industry	3	2.7	4	3.6	7	3.1
	Politics, policies	3	2.7	3	2.7	6	2.7
	Total	10	8.9	8	7.3	18	8.1
							
**Individual and group influences**								
	Experience with past vaccination	6	5.3	5	4.6	11	4.9
	Beliefs and attitudes about health and prevention	4	3.6	2	1.8	6	2.7
	Knowledge/awareness	40	35.4	43	39.1	83	37.2
	Health system and providers—trust and personal experience	3	2.7	5	4.6	8	3.6
	Risk/benefit (perceived, heuristic)	27	23.9	26	23.6	53	23.8
	Immunization as a social norm versus not needed/harmful	7	6.2	10	9.1	17	7.6
	Total	87	77.0	91	82.7	178	79.8
							
**Vaccine/vaccination specific issues**								
	Risk/Benefit (scientific evidence)	-	-	1	0.9	1	0.5
	Introduction of a new vaccine or new formulation	6	5.3	5	4.6	11	4.9
	Mode of administration	2	1.8	-	-	2	0.9
	Vaccination schedule	8	7.1	3	2.7	11	4.9
	Costs	-	-	1	0.9	1	0.9
	Role of healthcare professionals	-	-	1	0.9	1	0.9
	Total	16	14.6	11	10.0	27	12.1
							
	Grand Total	113	100.0	110	100.0	223	100.0

## Discussion

### Principal Findings

Overall, Facebook was a successful recruitment method for parents to complete a Web-based survey on vaccination. Out of the three advertisements posted in French and English, the English advertisements were the most successful with the highest CTR and subsequently lowest CPC and Advertisement A producing the highest CTR and lowest CPC. We were able to exceed our ideal sample size within a short timeframe, at low cost, and with one researcher running the campaign and data collection. The cost (Can $4,861), timeliness, and sample size of this study achieved comparable or better results than other recent health studies using targeted recruitment via Facebook [20-24,33]. For both the Web-based and RDD survey methods, data collection spanned the same time frame and individual surveys took approximately the same amount of time; however, the costs were 97% lower with Web-based recruitment. The quality of the data was evident with a rich pool of qualitative and quantitative data, a high completion rate, and little missing data. Although both monetary figures represent the total research costs, there are some notable differences as the Web-based recruitment costs also include incentives (no incentives were utilized in the RDD survey), but does not include the supplementary research costs associated with the use of an outside agency (eg, salaries and resources for implementation and deliverables). Notwithstanding, a typical RDD phone survey of 1000 participants might cost approximately Can $70,000 [34], and as demonstrated in this study, external contractual services would not necessarily be needed using Facebook targeted recruitment as it is a less labor-intensive process.

This study solely recruited from one social media platform, Facebook. However, respondents could have also been recruited from other social media platforms such as Twitter. Although Twitter users are typically younger and in childbearing/childrearing years [35], Twitter does not permit targeted recruitment via paid advertisement. Thus, it was considered as a supplementary sampling strategy should we not reach our pre-determined sample size of completed surveys via targeted recruitment on Facebook. Furthermore, Quach et al (2013) reported less success using a social network strategy (as opposed to a paid advertisement) in the recruitment of Canadian parents via Facebook and Twitter [36].

In both populations, the majority of the respondents were Canadian born, followed similar distribution patterns in terms of province/territory of residence, had mostly higher education levels and higher household income levels than the median total household income. We did not compare our data to census data as we were not trying to generalize to the Canadian population. In addition, census data is not available specifically for Canadian parents, our target population. The high response from residents of Alberta in the Web-based sample could be the result of higher engagement due to a large measles outbreak in Alberta that occurred in the month before our campaign launch [7]. The lower number of Québec responses was surprising as Québec has the second highest Facebook usage next to Ontario [27] and experienced a large measles outbreak in 2011. Moreover, we specifically targeted French Facebook users. Based on the lower success of our French campaigns, it is possible that the advertisements were not as attractive to French-speaking Québec users or that Québec users do not interact on the Internet in the same manner as Ontario users or that a higher percentage of the budget needed to be allocated to the French campaign to reach more French-speaking Facebook users.

The Web-based sample demographics differed because we recruited a majority of female respondents and a younger population with younger children compared to the RDD sample that had fairly equal representation of males and females, an older population (even if we assumed all of the unknowns were below 35 years), and older median age of the youngest child. As evidenced by the impression demographics, the Facebook campaign biased the recruitment toward a younger and female population, however the advertisements were intended to target parents with younger children as this would be the demographic interacting on the Internet and making decisions on childhood immunizations. Furthermore, Dubé et al (2012) reported no difference between mothers and fathers in intentions to vaccinate [37]. Combined, both methods produce the greatest spectrum of respondents; however, the Facebook campaign recruited more parents with young children at the most important stage of the vaccination process. Some of the differences we observed may be due to cohort effects, as vaccine hesitancy may have been increasing and been more prevalent in the younger parents recruited through Facebook.

According to our indicators, the Web-based strategy was successful in recruiting a higher number of vaccine-hesitant parents: more respondents perceived childhood immunizations to be not safe to moderately safe, more reported their youngest child’s vaccination status as not-up-to-date and more had difficulty in making the decision to vaccinate their youngest child. In addition, out of those reporting difficulty in the decision to vaccinate, more than half in the Web-based sample reported their youngest child’s vaccination status as not up-to-date. Moreover, one-fifth of the respondents who reported their child as not-up-to-date reported concerns over autism or sudden infant death syndrome as important reasons for deciding to not vaccinate their youngest child, even though it has been proven that neither disorder is associated with vaccination [38-40]. No significant contributions were observed when stratifying by age, sex, or parity; however, low numbers in some categories prevented reliable comparisons from being made. The factors associated with parental decisions to not vaccinate have been well studied [12,41,42], but no study has focused on vaccine-hesitant Canadian parents. We found that the main reasons reported for difficulty in decision-making were the inability to decipher or trust all the information available and the difficulties in weighing the risks and benefits of immunization with concerns over side effects and adverse effects. The contextual influence of media, social media, or other sources of communication may have played an important role in contributing to respondent concerns regarding their own knowledge or risk perception. However, this could not be further probed because of the inherent limitations of Web-based surveys.

### Limitations

As more people abandon landlines, the validity of traditional population telephone surveys is compromised with low response rates and potentially non-representative samples [43]. Representativeness and validity concerns are also relevant for Web-based surveys as research relies on the collection of self-reported data by self-selected participants [44]. However, there are more and more people on popular social media platforms such as Facebook and possibly different people than those reached by RDD. For example, active social media users may be mostly represented by educated females in higher income brackets [17,45,46], which is also the demographic most often looking for health information on the Internet [18]. Furthermore, active social media users may be potentially viewing an abundance of anti-vaccination sentiment on the Internet and may be the people that need to be reached most to combat vaccine hesitancy [47,48].

Both sampling techniques produced low response rates of 23%, which could produce biased samples. The reasons for the low response rate in the RDD sample include invalid numbers, unresolved callbacks, ineligibility, and refusals [25]. In the Web-based survey, there was no direct communication with the potential respondents; thus, it is not clear why certain Facebook users did not click on the advertisement or why those who clicked on the advertisement did not participate in the Web-based survey. This could be an important area for future study.

However, purposive Facebook targeted recruitment of self-selected respondents was not intended to provide a sample representative of the RDD sample or the Census population, but to determine whether we could recruit more “at risk” vaccine-hesitant parents as compared with the standard sampling technique. Reaching a higher proportion of vaccine-hesitant respondents proved successful; however, there are several inherent biases in using Facebook as a recruiting platform and in targeted sampling to self-selectors. For example, the low recruitment of male respondents could be the result of Facebook’s targeting criteria, the visuals, or the content of the advertisements. Selection bias is inevitable as Facebook identifies your desired target population, targets the most active and engaged users, and the number of impressions depends on factors such as the amount spent, the CTR, and market competition. However, for the purpose of our research, this proved to be a strength as this was the group we intended to target and would likely reach with any Web-based intervention. There is potential for volunteer bias and without a sampling frame we cannot calculate a true participation rate, nor can we characterize users who did not see the advertisement or did not engage. The timing of the advertisement (December) may have affected the type of respondents. However, this could not be verified without data from Facebook on who may be more likely to respond at different periods in time. Duplicate responses and gaming are also an important concern in Web-based recruitment [36]. Although difficult to prevent, safety measures as recommended in the Checklist for Reporting Results of Internet E-Surveys (CHERRIES) [49], were implemented to prevent and evaluate repeat respondents. Furthermore, it is possible that we attracted participants who were more likely to click on the advertisement because of strong views (anti or pro) on vaccination. However, based on our results, we were also able to reach an important proportion of participants who did not fall on the very extreme ends of the vaccination spectrum and reached a higher proportion of vaccine-hesitant parents when compared with the RDD.

As with any Web-based recruitment strategy, there are concerns about the “digital divide.” Statistics Canada recently reported that Canadians over the age of 65 years are responsible for the lag in Internet use in lower income households [17]. This population is not expected to represent a large proportion of our target population of vaccine-hesitant parents. In addition, the Web-based sample neglected to recruit many foreign-born parents or children. Yet, nearly 20% of Canadians were born outside of Canada. This might represent a significant bias that is difficult to quantify, as the Facebook activity of foreign-born residents is not known. Moreover, recent immigrants may not be a priority group to address for vaccine-hesitancy as they are more likely to arrive with immunity due to previous infections and more likely to become immunized as citizens [50,51]. Notwithstanding the inherent biases, we were able to obtain a large sample size for the recruitment period at a low cost, and we achieved a high survey completion rate with very little missing data. Furthermore, we reached younger Canadian mothers with younger children and more vaccine-hesitant parents when compared with the RDD sample.

### Conclusions

Targeted recruitment via Facebook was successful in reaching a population more likely to be engaging in health discussions on the Internet and making decisions on childhood immunizations. Thus, this recruitment strategy was superior to the RDD methodology in reaching “at-risk” vaccine-hesitant parents. Engaged respondents also provided us with insights into the most important determinants of vaccine hesitancy, providing valuable information in directing any future intervention efforts. With more Canadians abandoning landlines and interacting on the Internet with potential exposure to an abundance of anti-vaccination sentiment, popular social media platforms should be considered as part of any recruitment strategy or study on the determinants of vaccine-hesitant parents but also in the implementation of interventions to address these determinants. Future research should consider studies to investigate data reliability and to better examine the relative importance of contextual influences, such as the Internet, as determinants of vaccine hesitancy.

## Abbreviations

CPC: cost per click
CPM: cost per impression
CTR: click-through rate
IQR: interquartile range
IP: Internet Protocol
PHAC: Public Health Agency of Canada
RDD: random digit dialing
SAGE WG: Strategic Advisory Group of Experts Working Group
VPD: vaccine preventable disease
